# Effects of pressure-controlled and volume-controlled ventilation on respiratory mechanics and systemic stress response during prone position

**DOI:** 10.1186/s40064-016-3435-3

**Published:** 2016-10-10

**Authors:** Oznur Sen, Mefkur Bakan, Tarik Umutoglu, Nurdan Aydın, Mehmet Toptas, Ibrahim Akkoc

**Affiliations:** 1 Department of Anesthesiology and Reanimation, Ministry of Health Haseki Training and Research Hospital, Istanbul, Turkey; 2 Department of Anesthesiology and Reanimation, Faculty of Medicine, Bezmialem Vakif University, Vatan cad, 34093 Fatih, Istanbul Turkey

**Keywords:** Mechanical ventilation, Pressure controlled ventilation, Volume controlled ventilation, Prone position, Airway pressure, Stress response, Cortisol, Insulin

## Abstract

**Background:**

Prone position during general anesthesia for special surgical operations may be related with increased airway pressure, decreased pulmonary and thoracic compliance that may be explained by restriction of chest expansion and compression of abdomen. The optimum ventilation mode for anesthetized patients on prone position was not described and studies comparing volume-controlled ventilation (VCV) and pressure-controlled ventilation (PCV) during prone position are limited. We hypothesized that PCV instead of VCV during prone position could achieve lower airway pressures and reduce the systemic stress response. In this study, we aimed to compare the effects of PCV and VCV modes during prone position on respiratory mechanics, oxygenation, and hemodynamics, as well as blood cortisol and insulin levels, which has not been investigated before.

**Methods:**

Fifty-four ASA I-II patients, 18–70 years of age, who underwent percutaneous nephrolithotomy on prone position, were randomly selected to receive either the PCV (Group PC, n = 27) or VCV (Group VC, n = 27) under general anesthesia with sevoflurane and fentanyl. Blood sampling was made for baseline arterial blood gases (ABG), cortisol, insulin, and glucose levels. After anesthesia induction and endotracheal intubation, patients in Group PC were given pressure support to form 8 mL/kg tidal volume and patients in Group VC was maintained at 8 mL/kg tidal volume calculated using predicted body weight. All patients were maintained with 5 cmH_2_O PEEP. Respiratory parameters were recorded during supine and prone position. Assessment of ABG and sampling for cortisol, insulin and glucose levels were repeated during surgery and 60 min after extubation.

**Results:**

P-peak and P-plateau levels during supine and prone positions were significantly higher and P-mean and compliance levels during prone position were significantly lower in Group VC when compared with Group PC. Postoperative PaO_2_ level was significantly higher in Group PC compared with Group VC. Cortisol levels were increased with surgery in both groups (p < 0.05) and decreased to baseline levels in Group PC while remained high in Group VC in the early postoperative period. Cortisol levels were significantly higher in Group VC during surgery and in the early postoperative period compared with Group PC.

**Conclusion:**

When compared with VCV mode, PCV mode is associated with lower P-peak and P-plateau levels during both supine and prone positions, better oxygenation postoperatively, lower blood cortisol levels during surgery in prone position and in the early postoperative period. We concluded that PCV mode might be more appropriate in prone position during anesthesia.

## Background

Volume-controlled ventilation (VCV) that utilizes a constant flow to deliver a target tidal volume ensures minute ventilation, but reduced thoracic or lung compliance, increased airway resistance or active asynchronous exhalation of the patient may result in high airway pressures and increase the risk of ventilator-induced lung injury (Campbell and Davis 2002). Pressure-controlled ventilation (PCV), with its high and decelerating inspiratory flow, has faster tidal volume delivery and different gas distribution. The same tidal volume setting, delivered by PCV versus VCV, may result in a lower peak airway pressure and reduced risk of barotrauma, but variable tidal and minute volume may be generated (Campbell and Davis 2002; Wang et al. 2015; Jiang et al. 2016).

Prone position during general anesthesia for special surgical operations may be related with increased airway pressure, decreased pulmonary and thoracic compliance that may be explained by restriction of chest expansion and compression of abdomen (Tanskanen et al. 1997; Palmon et al. 1998). The optimum ventilation mode for anesthetized patients in prone position was not described and studies comparing VCV and PCV during prone position are limited (Choi et al. 2009; Jo et al. 2012; Kim et al. 2014).

We hypothesized that PCV instead of VCV during prone position could achieve lower airway pressures and reduce the systemic stress response. In this study, we aimed to compare the effects of PCV and VCV modes during prone position on respiratory mechanics, oxygenation, and hemodynamics, as well as blood cortisol and insulin levels, which has not been investigated before.

## Methods

This prospective randomized controlled study was approved by the Institutional Review Board of the Haseki Training and Research Hospital (No: 126, Date: 18.06.2014, Istanbul, Turkey). This study was carried out according to the principles of Declaration of Helsinki and all patients were asked for their signed and informed consent. Fifty-four patients, 18 to 70 years of age in the ASA I-II risk classification who underwent percutaneous nephrolithotomy in prone position were included in the study. Patients with morbid obesity (BMI exceeding 30 kg/m^2^), history of cardiac, pulmonary, hepato-renal, endocrine, cerebrovascular and neuromuscular diseases or thoracic surgery were excluded. Further, only those cases admitted to the operating theatre until 11:00 a.m. were included in this study. Patients were randomly selected, by opening sealed envelopes, to receive either PCV (Group PC: n = 27) or VCV (Group VC: n = 27) mode of ventilation during anesthesia.

On arrival at the operating room, standard monitoring was applied consisting of ECG, pulse oximetry and temperature. Intravenous midazolam 0.03 mg/kg was administered. After Allen test and local anesthetic infiltration, a cannula was placed to the radial artery and arterial pressure was monitorized. Blood sampling was made for baseline arterial blood gases (ABG) analysis and for cortisol, insulin and glucose levels to assess the systemic stress response.

Anesthesia was induced with propofol 2 mg/kg and fentanyl 2 μg/kg. Rocuronium 0.6 mg/kg was given to facilitate tracheal intubation (with a reinforced endotracheal tube). Anesthesia was maintained with 0.5–1.0 MAC of sevoflurane in a mixture of oxygen and air. Fentanyl 0.5–1 μg/kg was added to maintain systolic arterial pressure within ±20 % of the baseline value. Supplemental rocuronium was administered to maintain train-of-four (TOF) at 0 and 1. The ventilatory parameters were set as respiratory rate: 12 breaths/min, inspirium time/expirium time: 1/2, positive end-expiratory pressure (PEEP): 5 cmH_2_O and FiO_2_: 50 %, which were constant during anesthesia in both groups. Patients in Group PC were given pressure support to form 8 mL/kg tidal volume (pressure support level was adjusted to maintain the same tidal volume during anesthesia); while Group VC was maintained at 8 mL/kg tidal volume. Tidal volume was calculated using predicted body weight in both groups.

Heart rate, arterial pressures, end-tidal carbon dioxide pressure (EtCO_2_), peak, plateau and mean airway pressures (P-peak, P-plateau, P-mean respectively) and dynamic compliance (C-dyn) levels were recorded after intubation during supine position. All patients were transferred to classic prone position on a flat table. Head was in neutral position, the arms were raised beside the head, parallel chest rolls were placed from shoulder to hip on both sides, the legs were bent at the knees and all pressure points (forehead, elbow, knees, etc.) were padded. After surgery was started and at the 30th minute of prone position, respiratory and hemodynamic parameters were recorded again and sampling for ABG, cortisol, insulin and glucose levels were repeated.

For postoperative analgesia, paracetamol 1 g and tramadol 100 mg were administered and skin incisions were infiltrated with 10–15 ml of bupivacaine 0.5 % before closure. Anesthesia was maintained until the end of surgery. Neuromuscular blockade was antagonized with sugammadex 2 mg kg^−1^ and tracheal extubation was carried out when the patient was fully awake. Hemodynamic parameters were recorded and blood sampling for ABG, cortisol, insulin and glucose levels were repeated for the last time 60 min after extubation (without supplemental oxygen).

The primary outcome variable was P-peak levels during prone position. The sample size requirement was based on data from a previous study (Idem et al. 2009) in our institution, in which P-peak levels during prone position were 22.5 ± 5.1 cmH_2_O (in patients maintained at 8 mL/kg tidal volume with VCV and 5 cmH_2_O PEEP). Thus, at an alpha risk of 0.05, 27 patients per group would provide 90 % power to detect a 20 % reduction in P-peak levels.

For statistical analysis of the results Windows SPSS 15.0 program was used. Descriptive statistics were given in terms of numbers and percentages for categorical variables, and in terms of the mean, standard deviation and the median for the numerical variables. Comparison of two independent groups of variables was carried out using the Student T test when meeting the normal distribution criteria, or by the Mann–Whitney U test when these criteria were not met. Relationship between numerical variables was assessed by means of the Spearman Correlation Analysis. The differences between categorical variables were evaluated by the Chi square analysis. Statistical α (alpha) significance level was accepted with the ‘p’ value below 0.05.

## Results

Demographic parameters, and perioperative hemodynamic values of patients were closely comparable between groups (Table 1). Duration of surgery was 95 ± 32 min in Group PC and 92 ± 34 min in Group FOB, also comparable between groups.

**Table 1:** Demographic parameters and perioperative hemodynamic values

	Group PC (n = 27)	Group VC (n = 27)	*p* value
Age (year)	44.4 ± 12.3	41.1 ± 100	NS
Weight (kg)	78.9 ± 8.8	79.5 ± 10.3	NS
Gender (M/F)	20/7	22/5	NS
HR (beats/min)			
Pre	79.7 ± 10.4	76.5 ± 12.7	NS
Prone	78.7 ± 11.0	73.1 ± 9.4	NS
Post	76.1 ± 94	74.8 ± 8.2	NS
SAP (mmHg)			
Pre	133 ± 12.9	132 ± 14.5	NS
Prone	116 ± 13.1	117 ± 14.0	NS
Post	125 ± 11.9	123 ± 12.7	NS
MAP (mmHg)			
Pre	103 ± 13.4	103 ± 15.4	NS
Prone	93.9 ± 13.6	94.1 ± 8.5	NS
Post	92.8 ± 14.1	96.2 ± 13.6	NS

*HR* heart rate, SAP: systolic arterial pressure; MAP: mean arterial pressure; *PRE*: preoperative values; *PRONE*: values during prone position; *POST*: postoperative values; NS: not significant (*p* > 0.05)

Respiratory parameters during mechanical ventilation are shown in Table 2. Perioperative arterial blood gas analysis, cortisol, insulin, glucose and lactate levels are shown in Table 3. No statistically difference was found in EtCO_2_, PaCO_2_, pH, insulin and lactate levels between two groups. P-peak and P-plateau levels during supine and prone position were significantly higher in Group VC when compared with Group PC. P-mean and compliance levels during prone position were higher in Group PC when compared with Group VC. Postoperative PaO_2_ level was significantly higher in Group PC compared with Group VC.

**Table 2:** Respiratory parameters during mechanical ventilation

	Group PC (n = 27)	Group VC (n = 27)	p
EtCO_2_ (mmHg)			
I	32.6 ± 2.7	32.5 ± 2.9	NS
II	32.7 ± 3.0	34.1 ± 2.1	NS
P-peak (cmH_2_O)			
I	20.6 ± 2.6	22.2 ± 3.1	0.034
II	22.4 ± 2.1	23.9 ± 2.0	0.015
P-plateau (cmH_2_O)			
I	19.2 ± 2.9	21.3 ± 2.8	0.009
II	20.6 ± 2.5	22.0 ± 2.6	0.035
P-mean (cmH_2_O)			
I	11.2 ± 2.1	11.9 ± 2.1	NS
II	12.9 ± 1.4	11.6 ± 1.4	0.002
C-dyn (mL/cmH_2_O)			
I	44.1 ± 8.2	41.2 ± 11.1	NS
II	39.7 ± 7.8	31.9 ± 6.9	<0.001

*EtCO*_*2*_ end-tidal carbon dioxide, *P*-*peak* peak airway pressure, *P*-*plateau* plateau airway pressure, *P*-*mean* mean airway pressure, *C*-*dyn* dynamic compliance, *I* values after entubation, during supine position, *II* values during prone position, *NS* not significant

p < 0.05: statistically significant

**Table 3:** Perioperative arterial blood gas analysis and systemic stress response

	Group PC (n = 27)	Group VC (n = 27)	*p* value
PaO_2_ (mmHg)			
PRE	83.8 ± 8.5	84.0 ± 10.4	NS
PRONE	171.8 ± 47.3	156.0 ± 48.8	NS
POST	91.4 ± 8.0	83.5 ± 6.2	<0.001
PaCO_2_ (mmHg)			
PRE	39.3 ± 5.9	39.8 ± 6.7	NS
PRONE	36.5 ± 6.0	37.9 ± 5.6	NS
POST	39.4 ± 4.2	39.4 ± 4.4	NS
pH			
PRE	7.39 ± 0.05	7.37 ± 0.05	NS
PRONE	7.39 ± 0.05	7.37 ± 0.05	NS
POST	7.39 ± 0.05	7.38 ± 0.05	NS
Cortisol (mg/dL)			
PRE	11.45 ± 5.08	11.98 ± 4.76	NS
PRONE	17.77 ± 5.66	21.82 ± 6.61	0.016
POST	12.54 ± 6.45	22.92 ± 8.72	<0.001
Insulin (μU/mL)			
PRE	6.10 ± 3.33	4.81 ± 3.69	NS
PRONE	6.19 ± 5.01	5.65 ± 5.01	NS
POST	12.8 ± 15.9	11.1 ± 12.7	NS
Glucose (mg/dL)			
PRE	84.0 ± 14.5	79.5 ± 14.6	NS
PRONE	103.5 ± 25.1	105.8 ± 17.3	NS
POST	102.6 ± 19.2	113.8 ± 16.0	0.028
Lactate (mmol/L)			
PRE	2.28 ± 0.84	1.94 ± 0.83	NS
PRONE	2.59 ± 0.87	2.85 ± 0.70	NS
POST	2.08 ± 0.82	2.17 ± 0.72	NS

*PaO*_*2*_ arterial oxygen pressure, *PaCO*_*2*_ arterial carbon dioxide pressure, *PRE*: preoperative values, *PRONE* values during surgery in prone position, *POST* postoperative values, *NS* not significant (*p* > 0.05)

*p* < 0.05: statistically significant

Cortisol levels were increased with surgery in both groups (p < 0.05). Cortisol levels decreased to basal levels in Group PC and remained high in Group VC in the early postoperative period. Cortisol levels were significantly higher in Group VC during surgery and in the early postoperative period compared with Group PC. Postoperative glucose level was also significantly higher in Group VC compared with Group PC.

## Discussion

Major findings of the present study are PCV mode was associated with lower P-peak and P-plateau levels during both supine and prone positions, and higher P-mean levels during prone positions when compared with VCV mode. Mean airway pressure during inspiratory phase of respiration determines the recruitment of the collapsed alveoli and distribution of perfusion also it is a critic factor at gas exchange. PCV maintains higher P-mean levels, which may improve oxygenation. Postoperative PO_2_ levels, which were significantly higher in Group PC compared with Group VC, may indicate less alveolar de-recruitment. Cortisol levels were increased with surgery in both groups, while this increase was significantly higher in Group VC. The lower cortisol levels with PCV mode usage may be due to the deceleration flow form, lower P-peak, higher oxygenation and higher compliance levels which may result in lower incidence of atelectasis and pulmonary strain.

It has been showed that; elevated respiratory pressures could lead to acute lung injury (ALI) (Licker et al. 2003; Oeckler and Hubmayr 2007), higher P-peak levels could lead to lung edema after lobectomy (Van der Werff et al. 1997), and also minimal increases in P-peak can result the postoperative ALI risk (Fernandez-Perez et al. 2009). Studies reported that P-plateau lower than 35 cmH_2_0 was associated with lower incidence of death and barotrauma in patients (Amato et al. 1998; Boussarsar et al. 2002).

In our previous study (Sen et al. 2016), we have demonstrated that, when compared with VCV, PCV mode was associated with lower P-peak levels before and during pneumoperitoneum, better oxygenation and reduced systemic stress response postoperatively in patients having laparoscopic cholecystectomy. Our recent findings are mostly comparable with that study, but regarding insulin levels we could not demonstrate a significant difference between groups. Literature regarding systemic stress response during general anesthesia with different ventilation modes is limited with our two studies.

The meta-analysis that compared PCV and VCV modes during laparoscopic surgery by Wang et al. (2015) concluded that patients had PCV mode had lower P-peak and resistance accompanying higher compliance and P-mean levels. Although with subgroup analysis revealed the same results including morbid obese patients who underwent different kind of operations. Another meta-analysis by Jiang et al. (2016) included 27 trials with 1643 cases that compared PCV and VCV modes on different positions (supine, prone and lateral) and conditions (laparoscopic surgery, one lung ventilation, etc.); concluded that PCV mode was associated with increased oxygen index and decreased alveolo-arterial oxygen difference (A-aDO_2_). Subgroup analysis defining the effect of PCV mode on oxygenation concluded that patients having one-lung ventilation or laparoscopic surgery, and obese patients significantly benefit from the use of PCV, but patients on special positions did not. However, there is insufficient data comparing PCV and VCV modes during prone position.

Jo et al. (2012) found lower P-peak levels during PCV in both supine and prone positions when compared with VCV. Kim et al. (2014) compared the PCV and VCV modes during prone position in high-level spinal cord injury patients and found that P-peak increased after prone positioning in both groups, but this increase was significantly higher in VCV group compared with PCV group. In our study, compliance levels were lower in both groups in prone position when compared with supine. Compliance levels in prone position were found to be statistically higher in PCV group. Jo et al. (2012) found similar results like our study additionally they found higher compliance levels in both supine and prone positions during PCV ventilation.

## Conclusion

According to our findings, when compared to VCV mode, PCV mode is associated with lower P-peak and P-plateau levels during both supine and prone positions, better oxygenation postoperatively, lower blood cortisol levels during surgery in prone position and in the early postoperative period. We concluded that PCV mode might be more appropriate in prone position during anesthesia.
